# Prevalence of Diabetes and Hypertension among Hajj Pilgrims: A Systematic Review

**DOI:** 10.3390/ijerph18031155

**Published:** 2021-01-28

**Authors:** Saber Yezli, Abdulaziz Mushi, Yasir Almuzaini, Bander Balkhi, Yara Yassin, Anas Khan

**Affiliations:** 1 The Global Centre for Mass Gatherings Medicine, Ministry of Health, Riyadh 12341, Saudi Arabia; abhmashi@moh.gov.sa (A.M.); yalmuzini@moh.gov.sa (Y.A.); yyassin@moh.gov.sa (Y.Y.); khanaa@moh.gov.sa (A.K.); 2 Department of Clinical Pharmacy, College of Pharmacy, King Saud University, Riyadh 12372, Saudi Arabia; bbalkhi@KSU.EDU.SA; 3 Department of Emergency Medicine, College of Medicine, King Saud University, Riyadh 12372, Saudi Arabia

**Keywords:** mass gatherings, noncommunicable diseases, pilgrims, public health, diabetes, hypertension

## Abstract

The Hajj mass gathering is attended by over two million Muslims each year, many of whom are elderly and have underlying health conditions. Data on the number of pilgrims with health conditions would assist public health planning and improve health services delivery at the event. We carried out a systematic review of literature based on structured search in the MEDLINE/PubMed, SCOPUS and CINAHL databases, and the Preferred Reporting Items for Systematic Reviews and Meta-Analyses (PRISMA) guidelines, to estimate the prevalence of diabetes and hypertension among Hajj pilgrims. Twenty-six studies conducted between 1993 and 2018 with a total of 285,467 participants were included in the review. The weighted pooled prevalence rates of hypertension and diabetes among Hajj pilgrims in all included studies were 12.2% (95% CI: 12.0–12.3) and 5.0% (95% CI: 4.9–5.1), respectively. The reported prevalence of other underlying health conditions such as chronic respiratory, kidney or liver disease, cardiovascular disease, cancer and immune deficiency were generally low. Potentially a large number of pilgrims each Hajj have diabetes and/or hypertension and other underlying health conditions. Hajj could be a great opportunity to reduce the burden of these diseases within the over 180 countries participating in the event by identifying undiagnosed cases and optimizing patients’ knowledge and management of their conditions. Prospero registration number: CRD42020171082.

## 1. Introduction

Noncommunicable diseases (NCDs) are a significant global public health issue, being responsible for 41 million deaths each year (>73% of all deaths globally) and 53% of the 1.65 billion global years of life lost [1]. Population ageing allows the manifestation of many NCDs, which also result in a high prevalence of chronic disability [2]. Raised blood pressure (hypertension) and increased blood glucose are metabolic risk factors that can lead to NCDs. Hypertension is a major risk factor for stroke, cardiovascular disease (CVD), kidney disease and overall mortality [1,2,3]. Nonmodifiable risk factors for hypertension include a family history of hypertension, age > 65 years and coexisting diseases such as diabetes [4]. In terms of attributable deaths, hypertension is the leading metabolic risk factor globally, to which 19% of global deaths are attributed [5]. While it is a major health issue, fewer than 20% of people with hypertension have it under control [4]. Diabetes can lead to blindness, kidney failure, and lower limb amputation and has also emerged as a leading cause of disability globally [2]. The disease is also a major cause of mortality and leads to substantial economic loss. Diabetes resulted in an estimated 1.37 million deaths in 2017 and costed an estimated US $1.31 trillion in 2015 [1,6]. The true global burden of diabetes is probably underestimated due to death misclassification as well as underdiagnosis [7]. 

Hajj is an annual religious mass gathering that takes place in Makkah, the Kingdom of Saudi Arabia (KSA). It is attended by two to three million pilgrims from around the world and this number is expected to increase over the next few years [8]. Hajj pilgrims originate from over 180 different countries, two-thirds of them come from low- and middle-income countries. The Hajj population is characterized by a sizable elderly population (≈25% are >65 years old), many with underlying health conditions [9]. Physical and environmental stressors encountered during Hajj, in addition to changes in sleeping and nutrition habits, can lead to exacerbation of NCDs especially if pilgrims neglect to take their regular medications. As such, NCDs, such as CVDs and diabetes and related complications, are an important health risk during Hajj and a major cause of hospitalization and mortality among pilgrims [10,11,12,13,14]. 

Each year KSA dedicates a substantial amount of resources and funding into the planning and management of Hajj, including ensuring healthcare for pilgrims. KSA provides free healthcare for pilgrims during Hajj through numerous permanent and temporary hospitals, primary healthcare centers and other health facilities in the holy cities [15,16]. Information regarding underlying health conditions of prospective Hajj pilgrims would be extremely useful for optimum health service planning and delivery as well as for generating the appropriate health promotion campaigns prior to and during Hajj. However, currently there is no available health database for prospective Hajj pilgrims and there were no large-scale studies investigating the prevalence of underlying health conditions among pilgrims [17]. Hence, we systematically reviewed the literature to estimate the prevalence of two of the most commonly reported comorbidities among Hajj pilgrims; diabetes and hypertension. We also report the prevalence of other underlying health conditions documented in the identified studies. 

## 2. Method

### 2.1. Study Design

A systematic review was conducted to summarize the existing evidence concerning the prevalence of diabetes and hypertension among Hajj pilgrims according to the Preferred Reporting Items of Systematic reviews and Meta-Analysis (PRISMA) guidelines [18]. The review has been registered with International Prospective Register of Systematic Reviews (PROSPERO) as: CRD42020171082.

### 2.2. Data Sources and Search Strategy

MEDLINE/PubMed, SCOPUS and CINAHL databases were searched for available published articles in order to identify all potentially relevant publications prior to 1 January 2020. Additionally, the bibliographies of the eligible studies were scanned to include studies not identified through electronic database search. A search was conducted using a combination of keywords as follows: (“Hajj” OR “Pilgrim”) AND (“Hypertension” OR “Hypertensive” OR “Diabetes” OR “Diabetic’’). Wildcards, such as an asterisk, were used for all keywords.

### 2.3. Study Selection

**Figure 1:**
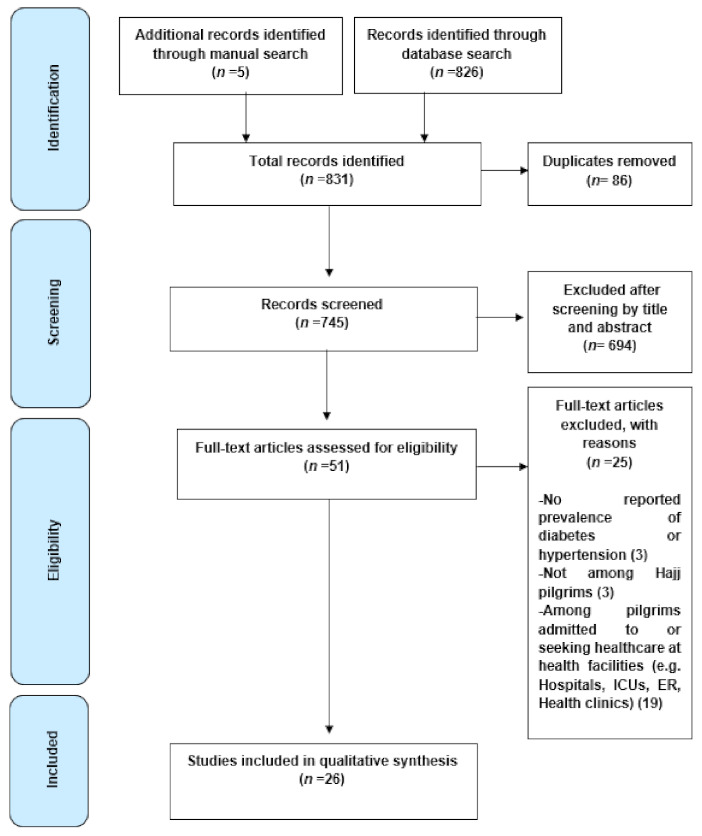
Preferred Reporting Items for Systematic Reviews and Meta-Analyses (PRISMA) study selection flow diagram.

Three reviewers (S.Y., B.B., A.M.) independently selected the studies for inclusion. The study protocol was defined and then reviewed by all three reviewers prior to starting the search for relevant studies. The reviewers independently conducted the search and then screened the title and abstract of each study for eligibility. In case the study could not be excluded based on the title and abstract, a full text was obtained for further analysis to determine its eligibility. Any discrepancy or disagreements between the reviewers regarding the included studies was resolved through consensus. The inclusion criteria were any study reporting primary data on the prevalence of diabetes and/or hypertension among the general Hajj population prior to 2020. Only studies published in English were considered. Studies that reported prevalence of disease in specific Hajj populations admitted to or seeking healthcare at health facilities (e.g., hospitals, intensive care units (ICUs), emergency rooms (ERs), health clinics) were excluded. The PRISMA statement was used to guide and report the search methodology (Figure 1). 

### 2.4. Data Extraction and Quality Management

Three authors (Y.A., A.M., Y.Y.) independently extracted the data from each study into an Excel database, while another author (S.Y.) arbitrated when discrepancy occurred. From each article data was extracted regarding the study design, study period, study population and demographics (sample size, age, gender, nationality), and prevalence of diabetes, hypertension and other underlying health conditions. Articles were evaluated to assess their quality using the Newcastle–Ottawa Scale (NOS) for cohort studies (http://www.ohri.ca/programs/clinical_epidemiology/oxford.asp) and a modified version for cross-sectional studies [19]. Two authors (Y.A., A.M.) independently evaluated the quality of the studies and any discrepancies were resolved through consensus. 

### 2.5. Data Synthesis and Statistical Analysis

Data were summarized in tables and synthesized in narrative form. To estimate the overall prevalence of hypertension and diabetes across all studies, we used the inverse variance weighting approach. The variance of the prevalence from each study was weighted by the inverse of its variance. A summary measure of the variance of the pooled variance was estimated by the inverse of the sum of the inverse variance of the estimate from each study. We calculated the 95% confidence interval for the pooled estimate using normal approximation formula. All statistical analyses were performed using R version 3.3.3 (Foundation for Statistical Computing, Vienna, Austria).

## 3. Results

### 3.1. Included Studies 

As seen from the PRISMA flowchart (Figure 1), the search strategy yielded 826 records and five additional papers were identified through manual search. After removing duplicates, 745 papers were screened by title and abstract and 51 full-text articles were reviewed for eligibility. Of these, 26 were deemed suitable for inclusion in the review and are presented in Table 1.

The reported mean age of the studied populations ranged from 33.5 to 66.3 years (20 studies) and the median age was around 60 years in four articles. Studies enrolled participants between the ages of 2 and 96 years, although most involved adult participants by design. Three studies purposefully enrolled only pilgrims aged <50 years, >50 years and ≥60 years, 13 studies enrolled adults ≥18 years, four studies did not have an age limit for inclusion, and the rest did not report on age criteria for enrollment. In general, most studies had a population with a large proportion of elderly pilgrims. In six studies, [29,36,41,43,44,45] 36.4–63.6% of the populations were aged ≥60 years and, in another seven investigations, [25,27,30,31,32,35,39] 15.4–39.3% of the populations were ≥65 years of age. Gender composition of the investigated populations was reported in 21 (80.7%) studies where the male:female ratio ranged from 0.6:1 to 2.7:1 (Table 1). 

Overall, the quality of the evidence was average for most studies according to the NOS score (Table 2). Cross-sectional studies were generally of lower quality compared to cohort studies. 

### 3.2. Prevalence of Diabetes and Hypertension among Hajj Pilgrims

The prevalence of hypertension among pilgrims was reported in 18 (69.2%) studies, four of which involved an actual medical examination of pilgrims. The prevalence of hypertension ranged between 5.1% among Iranian women under the age of 50 in 2005 [23] and 47% among Chinese pilgrims in 2017 [44]. In both studies, hypertension was investigated using clinical examination. In other studies among Iranian pilgrims, [21,28] prevalence of hypertension using clinical examination was less than 16%, while only 9.6% of Malaysian pilgrims in 2007 declared suffering from hypertension [26]. A study among pilgrims from 22 countries in Asia, Africa, Australia, North America, and Europe reported only 6.8% were hypertensive, [32] while the prevalence was 16.3% among pilgrims from Afghanistan, Bangladesh, Pakistan, Nigeria and South Africa [39]. In contrast, a study among pilgrims > 60 years old from 17 Arab speaking countries (Algeria, Bahrain, Egypt, Eritrea, Iraq, Jordan, Kuwait, Lebanon, Libya, Morocco, Oman, Palestine, Saudi Arabia, Sudan, Syria, Tunisia and Yemen) found a much higher prevalence of self-reported hypertension (42.6%) [42]. Among French pilgrims, 21.0–33.3% reported having hypertension (Table 1). The weighted pooled prevalence rate of hypertension among Hajj pilgrims in all included studies was 12.2% (95% CI: 12.0–12.3).

The prevalence of diabetes mellitus among Hajj pilgrims was reported in 25 (96.1%) studies, three of which involved an actual medical examination of pilgrims. The prevalence ranged from a low 1.6% among Omani pilgrims in 1996 [20] to nearly 40% among Chinese pilgrims in 2017 [44] A high rate of diabetes (32.1%) was also reported among Arab pilgrims > 60 years old in 2017 [42]. By comparison, low prevalence of diabetes (<10%) was found among Australian and Iranian pilgrims as well as pilgrims from KSA (79.1% of whom were Saudi nationals) [23,28,34]. Between 18.4% and 32.8% of French Hajj pilgrims self-reported being diabetic (Table 1). The weighted pooled prevalence rate of diabetes mellitus among Hajj pilgrims in all included studies was 5.0% (95% CI: 4.9–5.1).

### 3.3. Prevalence of Other Health Conditions among Hajj Pilgrims 

The prevalence of at least one underlying health condition among pilgrims was reported in 11 studies and ranged from 8.1% among pilgrims from Riyadh, KSA in 2003 [22] to 58.2% among French pilgrims in 2012 [37]. Other studies among French pilgrims conducted between 2006 and 2014 reported that 27.0–57.5% had at least one underlying health condition (Table 1). One study among pilgrims of varying nationalities reported that 27.7% had an underlying health condition [39].

Eight studies reported on the prevalence of chronic kidney disease among Hajj pilgrims. The prevalence was 0.3–2.5% among French pilgrims, [30,31,35,41,43,45]. 0.9% among Australian pilgrims [34] and 0.6% among pilgrims from five countries in Africa and Asia [39]. Chronic respiratory disease was reported in 17 studies with a prevalence ranging from 1.2% among pilgrims from various countries in 2015 [39] to 13.2% among French pilgrims in 2018 [43] Other studies among French Hajj pilgrims reported that 3.9–11.8% suffered from chronic respiratory disease (Table 1) while the prevalence among Australian pilgrims was 3.0% [34]. Chronic obstructive pulmonary diseases (COPD) was self-reported by 8.8% of Malaysian pilgrims in 2007, [26] while the prevalence of COPD and asthma was only 1.9% among 254,823 Iranian pilgrims investigated between 2004 and 2008 [28]. Similarly, only 1.6% of pilgrims traveling from Riyadh, KSA, in 2003 reported suffering from bronchial asthma [22].

CVD was not clearly defined in most studies included in this review. Nevertheless, 0.5#x2013;10.7% of pilgrims were reported to have chronic cardiac disease (Table 1). The lowest rate was reported among Malaysian pilgrims in 2007 [26] and the highest was among French pilgrims in 2018 [43] Another nine studies among French pilgrims reported that 3.4–9.4% had chronic cardiac disease, with most (77.8%) reporting levels >6%–<9.5% (Table 1). A study among Australian pilgrims between 2011 and 2013 reported that 3.3% had chronic cardiac disease [34] and a similar prevalence (3.7%) was found among Iranian pilgrims between 2004 and 2008 [28]. Another study among 4059 Iranian pilgrims > 50 years old examined in 1993 reported that 2.5% had ischemic heart diseases, 3.5% had heart failure and 3.6% had other CVDs [21]. The prevalence of CVDs among pilgrims from five countries (Afghanistan, Bangladesh, Pakistan, Nigeria and South Africa) was 4.4% in 2015 [39]. The same study found that 0.1% had a history of stroke, the same prevalence of stroke found among 195,949 Iranian pilgrims between 2005 and 2007 [28,39].

Cancer, immunodeficiency, chronic liver disease and neurological and psychological disorders were reported in a small proportion of Hajj pilgrims. Cancer was not reported among French pilgrims in 2013 [31] and 0.2% of Malaysian pilgrims in 2007 as well as pilgrims from Afghanistan, Bangladesh, Pakistan, Nigeria and South Africa in 2015 self-reported the condition [26,39]. In the latter study, 0.7% of pilgrims had chronic liver diseases but none were immune deficient [39]. Similarly, two studies among French pilgrims in 2013 and 2015 did not report immunodeficiency in any of the pilgrims investigated [31,45]. However, in other studies among French pilgrims, 0.6–3.3% reported being immunocompromised (Table 1). Chronic neurological disease was reported among 0.3% of Australian pilgrims in 2011–2013 [34] and 0.2% of Iranian pilgrims in 2004–2008 had dementia [28]. The latter study also reported that 1% of the 254,823 pilgrims investigated had psychiatric disorders [28].

Hyperlipidemia was reported in 15.3% of Arab pilgrims over the age of 60 in 2017 [42] and in 3.8% of Iranian pilgrims under 50 years old in 2005 [23]. Similarly, hypercholesterolemia was found in around 10% of French pilgrims in 2006 and 2007 [24,25]. Chronic sinusitis and chronic tonsillitis were reported in 1.9% and 1.6% of pilgrims from KSA respectively in 2003 [22] while 7.2% of Malaysian pilgrims in 2007 had allergic rhinitis [26]. Over a quarter of French pilgrims in 2007 had walking disability [24] and 18.8% of the 224,786 Iranian pilgrims investigated between 2005 and 2008 had musculoskeletal disease [28].

## 4. Discussion

We systematically reviewed the literature on the prevalence of diabetes and hypertension, two of the most commonly reported comorbidities among Hajj pilgrims. We found that an estimated 12.2% of Hajj pilgrims have hypertension and 5% are diabetic. Given that, currently, around 2.5 million pilgrims attend Hajj each year, it follows that in a given Hajj season, approximately 300,000 pilgrims have hypertension and 125,000 have diabetes. These figures may be an underestimation given that most studies involved self-reported conditions and many people are undiagnosed and are unaware they have these conditions [46,47,48] Moreover, as both the numbers of Hajj pilgrims and people with diabetes and hypertension worldwide are expected to significantly increase in the future, so is the number of pilgrims with these health conditions. This review also reports that a large proportion of pilgrims has underlying health conditions and confirms that diabetes and hypertension are by far the most commonly reported comorbidities. These results are in accordance with other large sample size reports among Hajj pilgrims [49,50]. For many pilgrims, the Hajj journey includes travel between different time zones, emersion in physically demanding religious rites, often in crowded settings and hot weather, as well as changes in diet and daily routines. These factors render the management of underlying health conditions during Hajj challenging and may lead to their aggravation. As such, pilgrims with underlying health conditions are over-represented among hospital and ICU admissions as well as deaths during the pilgrimage [49,50,51,52,53] 

The global prevalence of diabetes in 2019 was estimated to be 9.3%, [54] which is higher than the weight pooled prevalence found in our study. This is likely due to the fact that the two studies with the largest pilgrims’ sample size reported low rates of diabetes and were conducted over 10 years ago [20,28] Most of data relating to prevalence of diabetes in the review came from pilgrims of two countries, Iran and France. Among Iranian pilgrims in studies conducted between 2005 and 2008, a low (<6%) prevalence of diabetes was reported [23,28]. This is in line with the prevalence of diabetes among the adult Iranian population reported during the same period. For example, a national survey in Iran in 2005 found that 7.7% of adults aged 25–64 years had diabetes, 50% of whom were undiagnosed [48]. However, by 2011, the prevalence of diabetes among Iranians aged 25–70 years increased to 11.4% [55]. Interestingly, while conducted among small sample sizes, studies among French pilgrims reported much higher prevalence of diabase (18.4–32.8%). These levels are 2.5–6.5 times higher than the prevalence of diabetes reported among the adult population in France (5.1% in 2006–2007 and 7.4% in 2014–2016) [56,57] This discrepancy is likely due to the fact that the studies included in the review were mostly among older populations of individuals who were born in North Africa [24,25,27,29,33,35,36,38,40,41,43]. The prevalence of diabetes in France was shown to increase with age and is higher among North African immigrant population living in the country [57,58]. Fosse-Edorh et al. [58] reported that the prevalence of Type 2 diabetes was 14.0% in participants born in North Africa compared to 7.5% in those born in France. 

Worldwide, the number of adults with hypertension increased from 594 million in 1975 to 1.13 billion in 2015, where 25% of men and 20% of women had hypertension [4]. The increase was seen largely in low- and middle-income countries where most (66%) of people with hypertension live [4,59]. These countries are also where most of Hajj pilgrims originate from. The prevalence of hypertension among pilgrims was >20% in most studies reflecting global estimates [4]. However, two studies reported much high prevalence of hypertension. Zhang et al. [44] found that 47% of prospective adult Hajj pilgrims from Gansu province, China in 2017 had hypertension, while Alzahrani et al. [42] reported that 42.6% of pilgrims from 17 Arab speaking countries had the condition. Prevalence of hypertension in China varies between provinces, with some, including Gansu province, having a high proportion of hypertensive adults especially among older people [60,61,62]. A study among the adult population from Gansu province in China in 2013 reported that 36.7% had hypertension and only 37% of whom were aware of their condition [47]. Similarly, prevalence of hypertension in Arab countries is high especially among certain countries where levels of over 40% have been reported [63]. 

The Hajj journey presents specific challenges for pilgrims with diabetes. During the event, pilgrims are faced with physically demanding religious rites, changes in diets and habits including erratic eating schedules and delays in meal times, suboptimal hydration as well as hot temperatures, which may impact their physiology, absorption of insulin and interferes with its storage [12]. Poor compliance with treatment while preoccupied with Hajj rituals as well as lack of knowledge about self-management of diabetes in general and during Hajj specifically, are also common [46]. Hajj also involves walking substantial distances most undertaken with no protective footwear and sometimes barefoot [20,64]. As such, foot injuries are common during the pilgrimage increasing the risk of wound infections and complications, especially among people with diabetic neuropathy [36,64,65]. The above in addition to respirator trach infections, common among pilgrims, [35] can result in poor diabetes control, increasing the risk of hospital admission and mortality among diabetics during Hajj [51,52,66]. Although hospital admission and mortality directly due to diabetes-related complications are few compared to other causes; this number is on the increase and people with diabetes constitute a significant proportion of hospitalized pilgrims [12]. Among 140 pilgrims admitted to ICU during the 2004 Hajj, 25.7% had diabetes and 7.1% were admitted for diabetic ketoacidosis while a further 1.4% were admitted for hypoglycemia or uncontrolled diabetes [51]. Similarly, among 689 patients admitted to a tertiary care hospital in the 2005 Hajj, 220 (32%) had diabetes and 27 (4%) had diabetes-related complications [52]. 

Hypertension is also present in a large proportion of hospitalized pilgrims during Hajj [51,52]. Along with diabetes, hypertension is a major risk factor for the principal cause of death among pilgrims, CVD [67]. Almekhlafi et al. [68] reported that the incidence of stroke during the 2015 Hajj was 8.9/100,000. The most common risk factors were hypertension and diabetes found in 57% and 41% of the cases, respectively. While infectious causes may dominate hospital admission in Hajj, CVD has emerged as the main cause of both ICU admission and mortality during the event [14]. Among ICU admission in the 2004 Hajj, nearly 64% were due to CVD, especially myocardial infarction (25%) [51]. Hypertension was the main cause for 3% of the ICU admissions and a comorbidity in 26.4%. Just over 10% of patients died, 60% of whom were due to CVD. In 2002, CVD was the main cause of death among Hajj pilgrims (45.8%) and hypertension accounted for 2.7% of the total deaths [69]. A review of data from the Indian medical mission for three successive Hajj seasons (2014–2016), reported diabetes and hypertension as the main pre-existing conditions among pilgrims and over 70% of mortality was due to CVD [49]. Similarly, among the nearly 1.7 million Indonesian pilgrims who attended Hajj between 2004 and 2011, hypertension and diabetes were common and CVD contributed to 45–66% of deaths [50]. 

The burden of underlying health conditions other than diabetes and hypertension varies between countries, genders, age groups and over time. The global prevalence of chronic respiratory diseases in 2017 was around 7.1%, over 50% of which were COPD [70] Prevalence was highest among high-income countries and lowest in sub-Saharan Africa and South Asia. This is in accordance with our results, where prevalence ranged from 1.2% among pilgrims from Sub-Saharan Africa and South Asia to 13.2% among French pilgrims. The prevalence of other underlying health conditions among pilgrims reported in this review, including chronic kidney and liver diseases, CVDs, dyslipidemia and cancer, was relatively low compared to global estimates. For example, the estimated global prevalence of chronic kidney disease is between 11 to 13%, although prevalence is much higher (>20%) in those over 60 years old [71]. In 2008, the global rate of raised total cholesterol was 39% and over 50% of the population of high-income countries have elevated total cholesterol [72]. In 2015, there were an estimated 422.7 million cases of CVD and 6.11 million incident cases of chronic liver disease in 2017 [73,74]. In 2018, the total number of people who were alive within five years of a cancer diagnosis was estimated to be 43.8 million, with 18.1 million new cases [75]. Clearly, the global burden of the above conditions is significant and their prevalence in populations generally increases with age. Given that the Hajj population has a significant proportion of elderly individuals, it is expected that the prevalence of the above conditions is higher than what was found in this review. One possible explanation is that most studies included in the review used questionnaires to collect data and these conditions were self-reported. A large proportion of Hajj pilgrims have low level of educations and limited heath literacy [39]. Unlike diabetes and hypertension, which are more commonly known conditions, pilgrims may not easily recognize other health conditions even if they were diagnosed with them. 

Adequate health screening for prospective pilgrims with special attention to how well such chronic diseases have been managed by the individual is recommended and is in effect in certain countries [14,76]. Expending such health checkups to more countries may be challenging yet very useful not only in improving patients’ knowledge and management of their illnesses but also in identifying undiagnosed cases. A large proportion of people with diabetes, hypertension and other underlying health conditions are undiagnosed and awareness, treatment and control of these conditions are low, particularly in low- and middle-income countries [77,78]. Hence pre-Hajj health screenings could have a wider global health benefit. In any case, for pilgrims with pre-existing health conditions a pretravel consultation with a health professional is highly recommended to ensure they properly manage their conditions during the pilgrimages and bring sufficient supplies of their usual medications, consumables and monitoring instruments [12,38]. Ideally, data on pilgrims pre-existing conditions should be systematically collected and shared with the Saudi health authorities to support public health preparedness and planning for Hajj, ensure appropriate allocation of resources as well as improving response capabilities and health services delivery during the pilgrimage [17]. Such information would also assist in developing tailored and effective risk communication and health messages for pilgrims. Therefore, improving their knowledge of their conditions, medicine compliance, self-monitoring as well as taking measures to prevent negative health outcomes directly or indirectly related to their underlying health conditions. Health education before and during Hajj can also serve pilgrims post-Hajj for better knowledge and management of their conditions. 

This review has some limitations. First, we only included studies published in English; therefore, our review may suffer from language bias. Also, most studies had a low sample size, were of average quality and relied on self-reported underlying health conditions among pilgrims. Hence, potentially not reflecting the true prevalence of these conditions in the target populations. Finally, most studies were conducted among pilgrims from France or Iran, with only three reporting on prevalence among pilgrims from multiple countries. Future studies should consider random sampling of large populations of pilgrims from various nationalities and include clinical assessment to confirm the presence of these conditions and detect undiagnosed individuals. 

## 5. Conclusions

This systematic review indicates that potentially a large number of Hajj pilgrims suffer from diabetes and/or hypertension, rendering them a vulnerable population and potentially contributing to negative health outcomes during the event. Public health interventions to educate pilgrims on how best to manage their underlying health conditions in general and during Hajj should be a requirement, both at country of origin and in KSA. In addition, accurate data on the prevalence of these illnesses among prospective pilgrims is needed to ensure effective planning and delivery of health services as well as developing appropriate risk communication messages at the event. In general, the Hajj pilgrimage could be an ideal opportunity to reduce the burden of diabetes, hypertension and other health conditions within the over 180 countries participating in the event by identifying undiagnosed cases and optimizing patients’ knowledge and management of their conditions. 

## Figures and Tables

**Figure 2:**
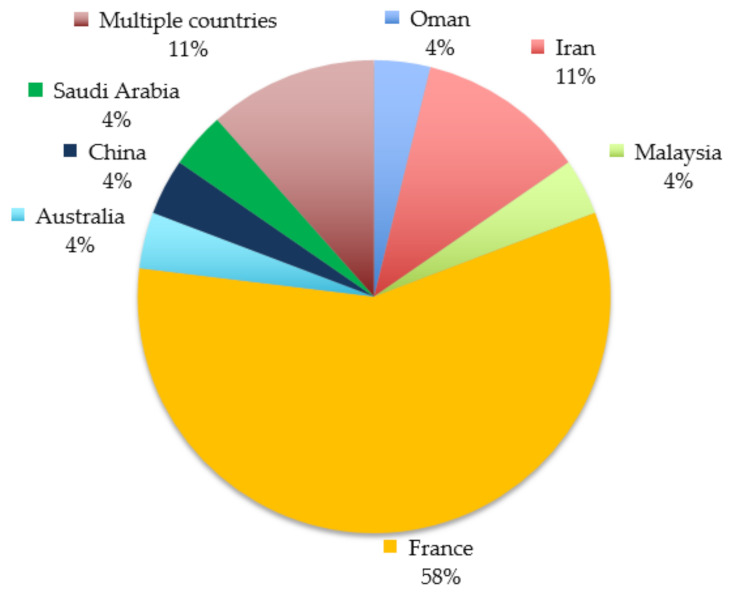
Geographical distribution of the studied populations among the 26 studies included in the systematic review.

**Table 1:** Summary of studies reporting on the prevalence of diabetes and/or hypertension among Hajj pilgrims.

Study				Study Population				Prevalence of UHCs n/N (%)			
Reference	Study Period	Study Design	Evaluation Method	Nationality	Sample Size	Age (Years)	Gender (Male: Female)	Diabetes	Hypertension	Other	At Least One UHC
Baomer and Elbushra. 1998 [20]	1996	CS	-	Oman	10,800	NR	NR	169/10800 (1.6%)	NR	-	NR
Afshin-Nia et al., 1999 [21]	1993	CS	C	Iran	4059	Mean = 60.6 ± 9.4	NR	NR	627/4059 (15.5%)	Heart failure: 143/4059 (3.5%) Ischemic heart disease: 102/4059 (2.5%) Other cardiovascular diseases: 147/4059 (3.6%)	NR
Choudhry et al., 2006 [22]	2003	CO	Q	KSA ^1^	1027	Mean = 33.5 ± 11.7	2.7:1	47/1027 (4.6%)	NR	Bronchial asthma: 16/1027 (1.6%) Chronic sinusitis: 19/1027 (1.9%) Chronic tonsillitis: 16/1027 (1.6%)	83/1027 (8.1%)
Azarpazhooh et al., 2008 [23]	2005	CO	C	Iran	896	NR	NR	29/896 (3.2%)	46/896 (5.1%)	Hyperlipidaemia: 34/896 (3.8%)	NR
Gautret et al., 2009 [24]	2007	CO	Q	France	545	Median = 61.0, range (02–87)	1.3:1	114/545 (21.0%)	113/545 (20.7%)	Chronic respiratory disease: 32/545 (6.0%) Chronic diarrhoea: 4/545 (1.0%) Walking disability: 134/545 (26%) Hypercholesterolemia: 52/545 (10.0%)	146/545 (27.0%)
Gautret et al., 2009 [25]	2006	CO	Q	France	580	Mean = 58.0, range (20–85)	1.3:1	132/580 (22.8%)	147/580 (25.3%)	Chronic respiratory disease: 23/580 (4.0%) Hypercholesterolemia: 60/580 (10.3%)	249/580 (43.0%)
Deris et al., 2010 [26]	2007	CS	Q	Malaysia	387	Mean = 50.3 ± 10.9	1.3:1	47/387 (12.1%)	37/387 (9.6%)	Cardiac disease: 2/387 (0.5%) Cancer: 1/387 (0.2%) COPD: 34/387 (8.8%) Allergic rhinitis: 28/387 (7.20%)	NR
Gautret et al., 2013 [27]	2010	CS	Q	France	523	Median = 60.0, range (19–86)	1.2:1	131/523 (25.0%)	133/523 (25.4%)	-	NR
Razavi et al., 2013 [28]	2004–2008	CO	C	Iran	254823	Mean = 51.0, range (15–95)	1.1:1	13471/254823 (5.3%)	30398/254823 (12.0%)	Cardiac disease: 9513/254823 (3.7%) Stroke: 249/195949 (0.1%) Asthma and COPD: 4976/254823 (1.9%) Musculoskeletal disease: 42324/224786 (18.8%) Psychiatric disorders: 2212/224786 (1.0%) Dementia: 438/254823 (0.2%)	NR
Benkouiten et al., 2013 [29]	2012	CO	Q	France	167	Mean = 59.3 ± 12.4, range (21–83)	0.6:1	46/167 (27.5%)	44/167 (26.3%)	Chronic respiratory disease: 13/167 (7.8%) Chronic cardiac disease: 12/167 (7.2%)	96/167 (57.5%)
Gautret et al., 2013 [30]	2013	CS	Q	France	360	Mean = 58.0, range (20–85)	1.1:1	83/360 (23.1%)	NR	Chronic kidney disease: 1/360 (0.3%) Chronic lung disease: 17/360 (4.7%) Chronic cardiac disease: 34/360 (9.4%) Cancer: 0/360 (0.0%) Immune deficiency: 6/360 (1.7%)	116/360 (32.2%)
Gautret et al., 2013 [31]	2013	CS	Q	France	114	Mean = 55.0, range (10–83)	NR	21/114 (18.4%)	NR	Chronic kidney disease: 1/114 (1.0%) Chronic lung disease: 7/114 (6.0%) Chronic cardiac disease: 6/114 (5.0%) Cancer: 0/114 (0.0%) Immune deficiency: 0/114 (0.0%)	33/114 (29.0%)
Memish et al., 2014 [32]	2013	CS	Q	Multiple countries ^2^	5235	Mean = 51.8, range (18–93)	1.2:1	21/160 (13.1%)	11/161 (6.8%)	-	
Gautret et al., 2014 [33]	2013	CO	Q	France	129	Mean = 61.7, range (34–85)	0.7:1	34/129 (26.4%)	43/129 (33.3%)	Chronic respiratory disease: 5/129 (3.9%) Chronic cardiac disease: 11/129 (8.5%)	68/129 (52.7%)
Tashani et al., 2014 [34]	2011–2013	CS	Q	Australia	954	Mean = 43.0 ± 13.1	1.9:1	86/954 (9.0%)	NR	Chronic kidney disease: 9/954 (0.9%) Chronic lung disease: 29/954 (3.0%) Chronic cardiac disease: 32/954 (3.3%) Chronic neurological disease: 3/954 (0.3%)	NR
Gautret et al., 2015 [35]	2012–2014	CO	Q	France	382	Mean = 60.6, range (22–85)	0.6:1	105/382 (27.5%)	115/382 (30.2%)	Chronic kidney disease: 1/382 (0.3%) Chronic respiratory disease: 29/382 (7.6%) Chronic cardiac disease: 32/382 (8.4%) Immune deficiency: 5/382 (1.3%)	210/382 (55.1%)
Sridhar et al., 2015 [36]	2013	CO	Q	France	129	Mean = 62.0	0.7:1	34/129 (26.4%)	43/129 (33.3%)	Chronic respiratory disease: 5/129 (3.9%) Chronic cardiac disease: 11/129 (8.5%)	NR
Verhoeven et al., 2015 [37]	2012	CO	Q	France	158	Mean = 59.6 ± 12.2	0.7:1	43/158 (27.2%)	NR	-	92/158 (58.2%)
Gagneux-Brunon et al., 2016 [38]	2013–2014	CS	Q	France	388	Mean = 52.9 ± 18.6	NR	78/364 (21.4%)	NR	-	126/364 (34.6%)
Yezli et al., 2017 [39]	2015	CS	Q	Multiple countries ^3^	1164	Mean = 54.5 ± 12.1, range (18–94)	2.6:1	125/1069 (11.7%)	174/1069 (16.3%)	Chronic kidney disease: 6/1069 (0.6%) Chronic respiratory disease: 13/1069 (1.2%) Chronic liver disease: 7/1069 (0.7%) Cardiovascular disease: 47/1069 (4.4%) Stroke: 1/1069 (0.1%) Cancer: 2/1069 (0.2%) Immune deficiency: 0/1069 (0%) Other: 36/1069 (3.4%)	296/1069 (27.7%)
Sow et al., 2018 [40]	2016	CO	Q	France	117	Mean = 61.0	0.9:1	31/117 (26.5%)	26/117 (22.2%)	Chronic respiratory disease: 11/117 (9.4%) Chronic cardiac disease: 4/117 (3.4%) Immune deficiency: 1/117 (0.9%)	NR
Hoang et al., 2019 [41]	2014–2017	CO	Q	France	485	Median = 61.5, range (21–96)	0.8:1	136/475 (28.6%)	140/475 (29.5%)	Chronic kidney disease: 5/475 (1.1%) Chronic respiratory disease: 56/475 (11.8%) Chronic cardiac disease: 32/475 (6.7%) Immune deficiency: 3/475 (0.6%)	NR
Alzahrani et al., 2019 [42]	2017	CS	Q	Multiple countries ^4^	340	Mean = 66.3 ± 5.9	1.2:1	109/340 (32.1%)	145/340 (42.6%)	Hyperlipidaemia: 52/340 (15.3%) Osteoarthritis, heart failure, allergies: 9/340 (2.6%)	NR
Hoang et al., 2019 [43]	2018	CO	Q	France	121	Median = 61.0, range (26–83)	0.9:1	31/121 (25.6%)	31/121 (25.6%)	Chronic kidney disease: 3/121 (2.5%) Chronic respiratory disease: 16/121 (13.2%) Chronic cardiac disease: 13/121 (10.7%) Immune deficiency: 4/121 (3.3%)	NR
Zhang et al., 2019 [44]	2017	CS	C	China	1465	Mean = 57.0 ± 9.4, range (30–70)	1.3:1	572/1465 (39.0%)	688/1465 (47.0%)	-	NR
Hoang et al., 2019 [45]	2015	CO	Q	France	119	Mean = 61.0	1.1:1	39/119 (32.8%)	NR	Chronic kidney disease: 1/119 (0.8%) Chronic respiratory disease: 12/119 (10.1%) Chronic cardiac disease: 8/119 (6.7%) Immune deficiency: 0/119 (0.0%)	NR

CS; cross-sectional, CO; Cohort, Q; questionnaire-based, C; clinical-based, NR; not reported, cohort, UHC; underlying health condition, COPD; chronic obstructive pulmonary diseases. ^1^ 79.1% were Saudi nationals. ^2^ Twenty-two countries in Asia, Africa, Australia, North America, and Europe. ^3^ Five countries (Afghanistan, Bangladesh, Pakistan, Nigeria and South Africa). ^4^ Seventeen Arab-speaking countries (Algeria, Bahrain, Egypt, Eritrea, Iraq, Jordan, Kuwait, Lebanon, Libya, Morocco, Oman, Palestine, Saudi Arabia, Sudan, Syria, Tunisia). Just over half (53.8%) of the included studies had a cohort design while the rest were cross-sectional. Only four studies involved medical examination of pilgrims while the rest were based on self-reported information through questionnaires. Studies were conducted between 1993 and 2018 with five studies running over multiple years. The total sample size was 285,467; however, over half (53.8%) of the studies included had a sample size <500. Only three studies were conducted among pilgrims of different nationalities, while the rest investigated pilgrims from specific countries, mainly France and Iran (Figure 2).

**Table 2:** Newcastle–Ottawa Scale (NOS) scores for the included studies.

**Cross-Sectional Studies**	**Selection** **(Max Five Stars)**	**Comparability** **(Max Two Stars)**	**Outcome/Exposure** **(Max Three Stars)**	**Total Score**
Baomer and Elbushra. 1998 [20]	**	-	*	3
Afshin-Nia et al., 1999 [21]	***	-	***	6
Deris et al., 2010 [26]	**	-	**	4
Gautret et al., 2013 [27]	**	-	**	4
Gautret et al., 2013 [30]	***	-	*	4
Gautret et al., 2013 [31]	*	-	*	2
Memish et al., 2014 [32]	***	-	***	6
Tashani et al., 2014 [34]	***	-	**	5
Gagneux-Brunon et al., 2016 [38]	***	-	**	5
Yezli et al., 2017 [39]	***	-	***	6
Alzahrani et al., 2019 [42]	****	-	**	6
Zhang et al., 2019 [44]	***	-	***	6
**Cohort Studies**	**Selection** **(Max Four Stars)**	**Comparability** **(Max Two Stars)**	**Outcome/Exposure** **(Max Three Stars)**	**Total Score**
Choudhry et al., 2006 [22]	**	-	**	4
Azarpazhooh et al., 2008 [23]	****	-	***	7
Gautret et al., 2009 [24]	**	-	**	4
Gautret et al., 2009 [25]	***	-	**	5
Razavi et al., 2013 [28]	****	-	***	7
Benkouiten et al., 2013 [29]	***	-	***	6
Gautret et al., 2014 [33]	***	-	**	5
Gautret et al., 2015 [35]	**	-	*	3
Sridhar et al., 2015 [36]	*	-	*	2
Verhoeven et al., 2015 [37]	*	-	***	4
Sow et al., 2018 [40]	***	-	***	6
Hoang et al., 2019 [41]	***	-	***	6
Hoang et al., 2019 [43]	***	-	***	6
Hoang et al., 2019 [45]	**	-	***	5

## Data Availability

The data presented in this study are available in the manuscript.
